# SKIM: A fast sketching strategy integrated with model’s dynamic-feedback for large-scale single-cell transcriptomic analysis

**DOI:** 10.1371/journal.pcbi.1014638

**Published:** 2026-08-10

**Authors:** Jiaxing Bai, Feng Zhou, Chongyang Tan, Yichun Gao, Yushuang He, Xiaobing Huang, Ying Wang

**Affiliations:** 1 Department of Automation, National Institute for Data Science in Health and Medicine, State Key Laboratory of Mariculture Breeding, Xiamen Key Laboratory of Big Data Intelligent Analysis and Decision, Xiamen University, Xiamen, Fujian, China; 2 National Institute for Data Science in Health and Medicine, Xiamen University, Xiamen, Fujian, China; 3 Department of Medical Oncology, Fuzhou First Hospital Affiliated with Fujian Medical University, Fuzhou, Fujian, China; Wuhan University, CHINA

## Abstract

The rapid expansion of single-cell RNA sequencing (scRNA-seq) datasets poses analytical challenges, including computational burden raised by increasing data scale, and imbalances in cell population sizes. Dataset sketching mitigates these issues by selecting representative subsets that preserve biological signals. Existing sketching methods rely on geometric distances between transcriptomic expression profiles to select representative cells that cover the expression space. While effective at capturing global expression patterns, these methods are insensitive to local expression variations that encode multiscale transcriptomic differences. Moreover, by prioritizing maximal coverage of the expression space, these methods overselect peripheral cells, including rare and abnormal cells. To overcome these limitations, we propose SKIM, a fast SKetching strategy integrated with model’s dynamic-feedback for large-scale single-cell transcriptomic analysis. Dynamic-feedback is defined as the sequence of reconstruction losses for each cell across training epochs, reflecting how the model progressively learns the transcriptomic expression profile. During training, as the model captures features from global expression patterns to local variations, dynamic-feedback from loss trajectories provides comprehensive multiscale views of the variation between cells. Based on dynamic-feedback, SKIM identifies and removes abnormal cells exhibiting unstable feedback patterns, and constructs sketches through clustering and size-aware sampling in the dynamic-feedback space, reducing data size while balancing cell populations. Across nine benchmark datasets, SKIM outperforms four state-of-the-art sketching methods in cell type annotation, data integration, bulk RNA-seq deconvolution, and developmental trajectory inference. Moreover, SKIM demonstrates high computational efficiency, achieving over a 20-fold speedup compared with existing methods when generating a 10% sketch from 216,611 cells. Overall, SKIM provides an efficient framework for large-scale dataset sketching that effectively retains critical biological signals while reducing computational burden.

## Author summary

The rapid growth of single-cell RNA sequencing (scRNA-seq) datasets has created major computational burden and severe imbalances in cell population sizes. Similar to selecting key frames that summarize an entire film, dataset sketching selects a small set of representative cells from large-scale single-cell datasets while preserving key biological signals. Unlike traditional methods based on geometric distances between transcriptomic expression profiles, we introduce SKIM, a fast sketching strategy integrated with dynamic-feedback, defined as the sequence of reconstruction losses for each cell across training epochs. Dynamic-feedback characterizes each cell from multiple perspectives across different epochs, thereby capturing features from global expression patterns to local variations. Based on dynamic-feedback, SKIM identifies and removes abnormal cells exhibiting unstable feedback patterns, and constructs sketches through clustering and size-aware sampling in the dynamic-feedback space, reducing data size while balancing cell populations. SKIM is computationally efficient, achieving over a 20-fold speedup over existing methods when generating a 10% sketch from a dataset of 216,611 cells. Across four downstream analyses, including cell type annotation, data integration, developmental trajectory inference, and bulk RNA-seq deconvolution, SKIM preserves sufficient biological information to enable accurate and reliable analyses, while substantially reducing dataset size.

## Introduction

Single-cell RNA sequencing (scRNA-seq) technologies have revolutionized our understanding of cell states and functions across tissues, developmental stages, and disease contexts [1,2]. However, as the number of profiled cells rapidly increases, downstream analyses face major challenges, including substantial computational burden and the pronounced imbalance in cell numbers across distinct cellular populations, which cause dominant populations to obscure signals from rare but biologically important cell states [3–5]. Here, cellular populations refer to groups of cells sharing similar transcriptomic expression profiles and representing specific cell types or states.

Dataset sketching has emerged as a computational strategy to address this challenge by constructing representative subsets of scRNA-seq data, referred to as “sketches,” which preserve the critical biological information inherent in the full dataset, including signals from dominant and rare populations, while substantially reducing data size and accelerating downstream analyses [6]. Existing methods, including GeoSketch [6], Sphetcher [7], Hopper [8], and scSampler [9], rely on geometric distances computed from transcriptomic expression profiles. Specifically, GeoSketch divides the high-dimensional expression space into equally sized grid boxes and selects cells from each box to ensure comprehensive representation of the space. Building on this idea, Sphetcher adopts a similar grid-based partitioning strategy but improves upon it by using spherical regions instead of boxes, allowing for better capture of radial variability in cell distributions. In contrast, Hopper applies an iterative farthest-first selection approach, choosing cells that are maximally distant from those already selected, thereby promoting broad coverage of the expression space. Similarly, scSampler optimizes cell selection to maximize the minimum pairwise distance among selected cells, ensuring that the resulting sketch is highly representative of the overall dataset. These sketching methods rely on geometric distances computed from transcriptomic expression profiles to achieve broad coverage of expression space for sketch construction. While effective at capturing global expression patterns, these methods are insensitive to local variations that encode multiscale transcriptomic differences. In addition, by prioritizing maximal coverage of the expression space, these methods tend to overselect peripheral cells, which may include biologically rare populations or abnormal cells, reducing the robustness and biological representativeness of the resulting sketches. Recently, scValue [10] introduced a classification-guided strategy for selecting cells based on known cell type labels. However, the applicability of scValue is limited by the frequent lack of comprehensive cell type annotations in many scRNA-seq datasets.

To address these limitations, a self-supervised framework that leverages the model’s dynamic-feedback is used to guide dataset sketching. Dynamic-feedback is defined as the sequence of reconstruction losses for each cell across multiple training epochs, reflecting how the model progressively learns the transcriptomic expression profile. During training, as the model captures features from global expression patterns to local variations, dynamic-feedback from loss trajectories provides comprehensive multiscale views of the variation between cells. Since cells with highly consistent feedback convey redundant information that potentially causes dominant populations to be overemphasized, this redundancy is mitigated through clustering-based sampling that adjusts selection according to cluster size. Furthermore, the framework effectively distinguishes rare from abnormal cells. Rare cells, despite their low abundance, maintain coherent expression patterns and produce stable feedback trajectories. In contrast, abnormal cells remain unpredictable throughout training, generating unstable dynamic-feedback trajectories that allow them to be reliably identified and excluded.

Motivated by this principle, we propose SKIM, a fast SKetching strategy Integrated with Model’s dynamic-feedback for large-scale single-cell transcriptomic analysis. SKIM derives dynamic-feedback to characterize each cell across multiple training epochs, capturing both global expression patterns and local variations. Abnormal cells are identified based on dynamic-feedback and removed, reducing their potential to distort the sketches. The remaining cells are clustered within the feedback space, and a size-aware sampling strategy is applied to eliminate redundancy and generate diverse, informative, and representative dataset sketches. Therefore, SKIM efficiently produces high-quality sketches that preserve key biological signals.

Across nine benchmark datasets, SKIM consistently outperforms four state-of-the-art sketching methods, achieving substantial dataset reduction while maintaining downstream performance in cell type annotation, data integration, developmental trajectory inference, and bulk RNA-seq deconvolution. In addition, SKIM demonstrates high computational efficiency, achieving more than a 20-fold speedup over existing methods when generating a 10% sketch from 216,611 cells. Overall, SKIM provides an efficient sketching framework that alleviates computational burden while preserving analytical accuracy in large-scale single-cell transcriptomic studies.

## Results

To evaluate the utility of sketching methods for accelerating downstream single-cell RNA-seq analyses while preserving biologically meaningful information, SKIM was benchmarked against four baseline methods across multiple common downstream tasks. Specifically, the four baseline methods are as follows: GeoSketch, which divides the high-dimensional expression space into equally sized grid boxes and selects cells from each box to ensure comprehensive spatial coverage; Sphetcher, which replaces grid boxes with spherical regions to better capture radial variability in cell distributions; Hopper, which employs a distance-based farthest-first traversal to iteratively select cells that maximize their distance from previously chosen ones; and scSampler, which directly optimizes for maximal pairwise distances among selected cells to prioritize the sampling of highly diverse cells. All baseline methods were applied with their recommended default parameters. For SKIM, the hyperparameter settings and detailed preprocessing are provided in S1 Table.

For all comparative experiments, sketching methods were applied to a shared PCA-reduced representation of the same input data to ensure consistency across methods. After standard preprocessing, including normalization, log-transformation when required, and highly variable gene selection, a 50-dimensional PCA representation was computed for each dataset and used as the common input for SKIM and all baseline methods. The baseline methods were then executed following the recommended or method-compatible procedures of their corresponding tools.

As summarized in Table 1, nine datasets were used to benchmark SKIM against four baseline methods across multiple common downstream tasks. For cell type annotation, the PBMC and CxG_min datasets were used to evaluate whether sketches preserve sufficient biological information to accurately classify cells into their ground-truth categories, reflecting the retention of cell type identity. The cross-dataset cell type annotation task used the Colon and Gut datasets to assess sketch performance across different biological conditions, testing whether cell type identity is maintained despite batch effects. For data integration, the ICA_cord_blood, ICA_bone_marrow and Mammary_Gland datasets examined whether sketches preserve the global structure of the original data in a manner that supports effective downstream integration, as quantified by the degree of batch mixing in a shared latent space. Bulk RNA-seq deconvolution, performed on the T&ILC dataset, evaluated whether representative transcriptomic expression profiles are maintained by comparing deconvolution accuracy between full and sketched reference datasets. Finally, developmental trajectory inference on the Hematopoiesis dataset assessed whether sketches retain diverse cell populations, including transitional cells and other rare states critical for reconstructing continuous differentiation paths, by comparing trajectories inferred from sketches with those obtained from the full dataset. Detailed descriptions of each dataset can be found in S2 Text.

**Table 1:** Overview of the nine scRNA-seq datasets included in this study.

Dataset	Cell number	Cell type	Usage
PBMC [11]	30,495	10	Cell Type Annotation
CxG_min [12]	65,536	164	Cell Type Annotation
Colon [13]	41,650	25	Cross-Dataset Cell Type Annotation
Gut [14]	428,469	134	Cross-Dataset Cell Type Annotation
ica_cord_blood [6]	384,000	18	Data Integration
ica_bone_marrow [6]	378,000	23	Data Integration
T&ILC [15]	216,611	16	Bulk RNA-seq Deconvolution
Hematopoiesis [16]	67,568	8	Developmental Trajectory Inference
Mammary_Gland [17]	15,577	7	Data Integration

In addition, computational efficiency and distributional consistency were evaluated to assess runtime and the extent to which sketches preserve the full data distribution, as measured by the Wasserstein distance [18]. Finally, to examine the impact of filtering abnormal cells, ablation experiments were performed comparing sketches generated with varying filtering ratios to those without filtering. The influence of clustering granularity on sketch quality was also assessed.

All experiments were conducted on a server running Ubuntu 24.04.2 LTS, equipped with two Intel Xeon Silver 4214 CPUs @ 2.20 GHz (48 logical CPUs total) and 566 GiB RAM. No specialized hardware acceleration beyond this shared computational environment was used for either SKIM or the baseline methods.

### Evaluating the ability of sketches to preserve cell-type identity in cell type annotation tasks

To assess the effectiveness of sketching methods in preserving cell type identity, two cell type annotation benchmark datasets were analyzed: PBMC and CxG_min. For PBMC, training data were derived from Seq-Well, inDrops, and Drop-seq platforms, whereas data from the 10x Chromium platform served as the test set. For CxG_min, training and test partitions followed the definitions provided in scTab [12]. Sketches were generated from the training sets at sampling ratios ranging from 2% to 10%, and each experiment was repeated five times to assess stability. Annotation performance was evaluated using ACTINN [19] and CellTypist [15], where each classifier was trained on each sketch and tested against ground-truth test set labels. This setup quantitatively measures how effectively each sketching method preserves cell type identity across datasets.

**Fig 1:**
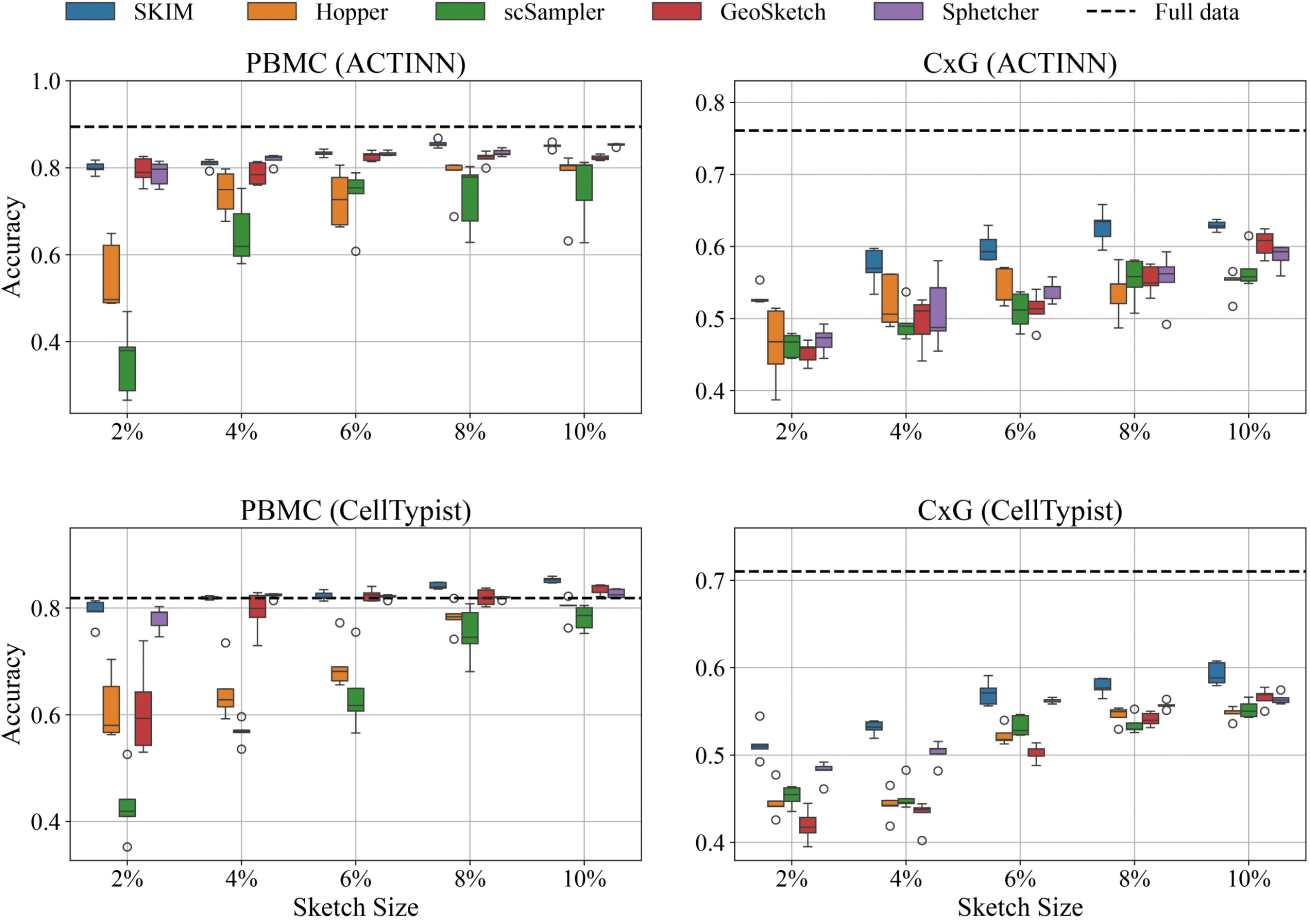
Comparison of annotation accuracy across sketching methods on the PBMC and CxG_min datasets, evaluated using ACTINN and CellTypist classifiers under varying sampling ratios.

Across both datasets, SKIM maintained the highest median classification accuracy in most settings and demonstrated stable performance across repeated runs (Fig 1). This strong performance can be attributed to SKIM’s sketching strategy based on dynamic-feedback, which enables the selection of cells that are informative for preserving cell type identity. Existing sketching methods, which rely on distances computed from transcriptomic expression profiles, are susceptible to the influence of abnormal cells. As a result, their performance was less stable and less accurate in our benchmarks. For instance, Hopper and scSampler yielded notably decreased accuracy on the PBMC dataset at the 2% sampling ratio, illustrating their tendency to produce unreliable sketches under more aggressive compression. Sphetcher performed well on PBMC but showed markedly reduced accuracy on CxG_min, suggesting limited generalization across datasets with differing levels of heterogeneity. Notably, on the PBMC dataset using CellTypist, SKIM achieved accuracy nearly identical to that of the full PBMC dataset (0.819) at a 4% sampling ratio (0.820), and at a 10% sampling ratio, it even surpassed the full dataset. Detailed results can be found in S2-S5 Tables.

These results demonstrate that SKIM generated sketches that effectively retained the discriminative features required for accurate cell type classification, providing both robustness and fidelity in downstream analyses compared with existing sketching strategies.

### Evaluating the ability of sketches to preserve cell-type identity in cross-dataset cell type annotation tasks

To further evaluate the ability of sketching methods to preserve cell type identity under more challenging conditions, their performance was assessed in a cross-dataset annotation setting, where batch effects create substantial distributional shifts between the reference and the query dataset. The Gut dataset, containing high-quality manually curated cell type labels, served as the reference dataset, while the Colon dataset, with a partially divergent annotation scheme, was used as the query dataset. Reference sketches were generated with SKIM and four baseline methods at a 1% sampling ratio. For each sketch, we trained a CellTypist classifier on the reference dataset and transferred the learned annotations to selected T cells in the query dataset. Prediction performance was assessed using confusion matrices, yielding one heatmap per sketching method.

**Fig 2:**
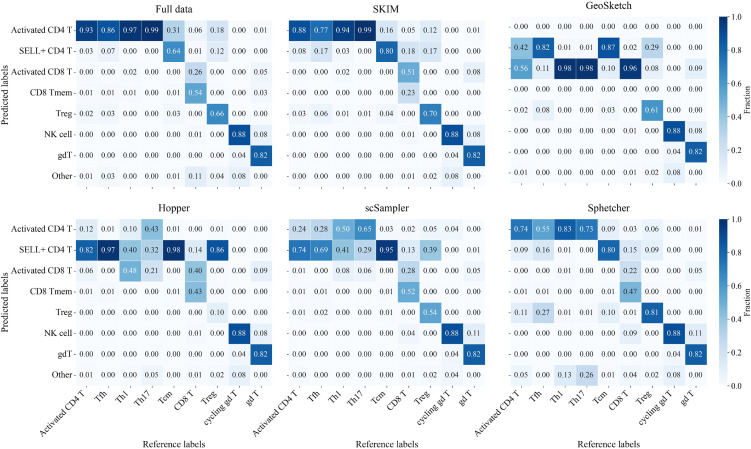
Confusion matrices comparing predicted and true cell type labels in the Colon query dataset using CellTypist trained on either a 1% sketch or the full Gut reference dataset.

As shown in Fig 2, the heatmap corresponding to SKIM exhibited the highest concordance with the predictions obtained from the full dataset with CellTypist, accurately preserving cell type identity. In contrast, heatmaps generated by GeoSketch, Hopper, scSampler, and Sphetcher displayed method-specific biases: GeoSketch tended to overassign cells to Activated CD8 T, Hopper favored SELL⁺ CD4 T, and although scSampler and Sphetcher mitigated some of these deviations, their overall concordance with the ground truth remained lower than that of SKIM. SKIM’s superior performance can be attributed to the sketching strategy based on dynamic-feedback, which emphasizes cells that contribute informative expression signals relevant to cell identity. By retaining such representative cells in the reference sketches, SKIM enables more accurate and consistent cross-dataset cell type annotation.

### Evaluating the ability of sketches to preserve global cellular structure in data integration task

To evaluate the ability of sketching strategies to accelerate single-cell RNA-seq data integration while preserving global cellular structure, two datasets from the Immune Cell Atlas (ICA) project, ICA_cord_blood and ICA_bone_marrow, were analyzed. Both datasets profiled immune-related cells but originated from different tissues, introducing both biological differences and potential technical variation. Sketches of each dataset were generated using GeoSketch, Hopper, scSampler, Sphetcher, and SKIM at a 1% sampling ratio. Integration models were trained on these sketches using Scanorama [20], and the learned transformations were applied to project the full datasets into a shared embedding. Integration performance was quantified using both the integration Local Inverse Simpson’s Index (iLISI), which measures batch mixing, and the cell-type Average Silhouette Width (ASW_label) from the scIB benchmark framework [21], which evaluates biological conservation by assessing the separation of annotated cell types. Higher iLISI indicates better batch mixing, whereas higher ASW_label indicates better preservation of cell-type identity.

**Fig 3:**
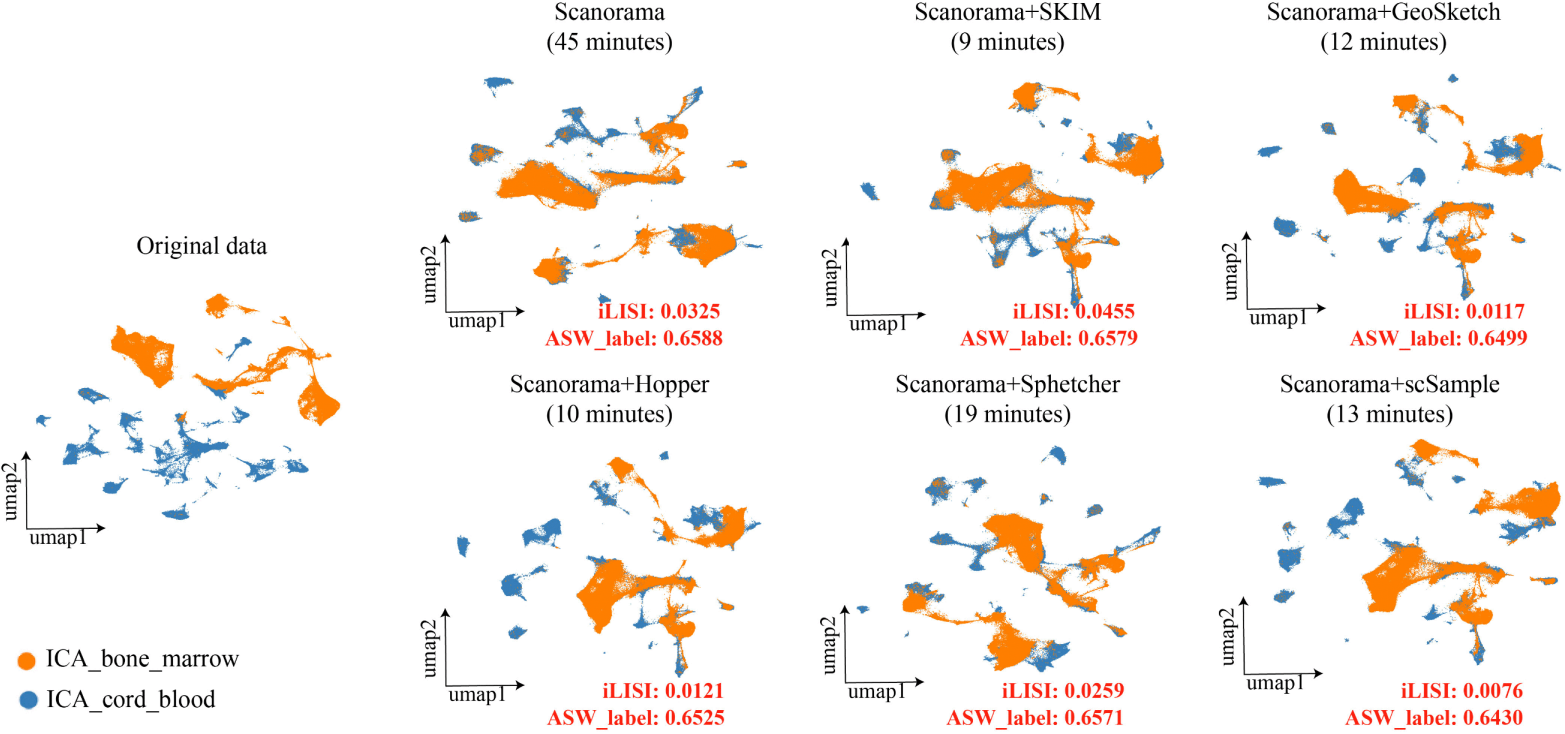
Integration performance across sketching methods on ICA_cord_blood and ICA_bone_marrow datasets, evaluated using UMAP embeddings and iLISI scores at a 1% sampling ratio.

As shown in Fig 3, SKIM achieved the highest iLISI score, outperforming all other sketching methods and even surpassing the integration performance obtained using the full dataset, while maintaining an ASW_label score comparable to that of the full dataset. Detailed iLISI and ASW_label results are provided in S6 Table. This improvement can be attributed to SKIM’s clustering-based, size-aware sampling strategy in the dynamic-feedback space, which captures global and local transcriptomic variation to selectively retain cells that make informative and non-redundant contributions, including transitional and functionally specialized cells. By preserving these biologically important populations, SKIM emphasizes key signals while maintaining the global structure of the dataset. Beyond enhancing integration quality, SKIM also substantially improves computational efficiency. The entire integration process, including sketch construction and integration with Scanorama, required only 9 minutes, with the sketching step taking just 6.8 seconds, compared with roughly 45 minutes for the full dataset, achieving more than a fivefold speedup.

In addition, to further evaluate the generalizability of SKIM in data integration, we added a same-tissue cross-technology benchmark using two mouse Mammary_Gland datasets [17] and evaluated SKIM using another widely used integration method, Harmony [22]. As shown in S6 and S7 Tables, SKIM showed competitive integration performance across different dataset settings and integration methods, supporting its broader applicability for sketch-assisted single-cell data integration.

### Evaluating the ability of sketches to extract representative transcriptomic expression profiles in bulk RNA-seq deconvolution task

To evaluate the ability of sketching strategies to extract representative transcriptomic expression profiles for bulk RNA-seq deconvolution, the T&ILC dataset comprising 216,611 cells from 12 human donors was analyzed. Expression matrices from two donors (A29 and A31) served as the full reference dataset, while the remaining ten donors were aggregated individually to generate pseudobulk samples with known ground-truth cell-type proportions. Reference sketches were generated using SKIM, GeoSketch, Hopper, Sphetcher, and scSampler at a 1% sampling ratio, and deconvolution accuracy was assessed using MuSiC [23]. Performance was quantified using mean absolute error (MAE), root mean squared error (RMSE), and average Pearson correlation between inferred and true cell-type proportions.

As shown in Table 2, SKIM achieved the lowest MAE (0.0327) and RMSE (0.0434) and the highest correlation (0.7256), even surpassing the full reference dataset. Among the baselines, Hopper and scSampler performed relatively well, GeoSketch yielded intermediate results, and Sphetcher performed the poorest, particularly in terms of correlation (0.4735). Scatter plots comparing inferred and true cell-type proportions (S1 Fig) further confirmed that SKIM most accurately recovered bulk composition across cell types. SKIM effectively selects representative cells capturing both global and local transcriptomic variation, enabling more accurate bulk deconvolution than other methods. These results highlight the advantage of SKIM for constructing compact yet biologically representative single-cell references in bulk RNA-seq analyses.

**Table 2:** Performance of bulk RNA-seq deconvolution using single-cell references generated by different sketching methods, evaluated by MAE, RMSE, and Pearson Correlation.

Method	MAE	RMSE	Correlation
Full	0.0473	0.0646	0.5629
SKIM	0.0327	0.0434	0.7256
GeoSketch	0.0392	0.0513	0.6083
Hopper	0.0381	0.0481	0.6169
Sphetcher	0.0427	0.0580	0.4735
scSampler	0.0361	0.0458	0.6653

### Evaluating the ability of sketches to preserve rare transitional cells in developmental trajectory inference task

The ability of sketching methods to retain diverse cell populations, particularly rare transitional cells, which are critical for reconstructing continuous differentiation paths, was evaluated through developmental trajectory inference using CellRank2 [16] on the Hematopoiesis dataset. Sketches were generated by sampling 20% of cells to balance retention of subtle developmental continua with computational efficiency. Trajectories inferred from the full dataset served as the reference for comparison.

**Fig 4:**
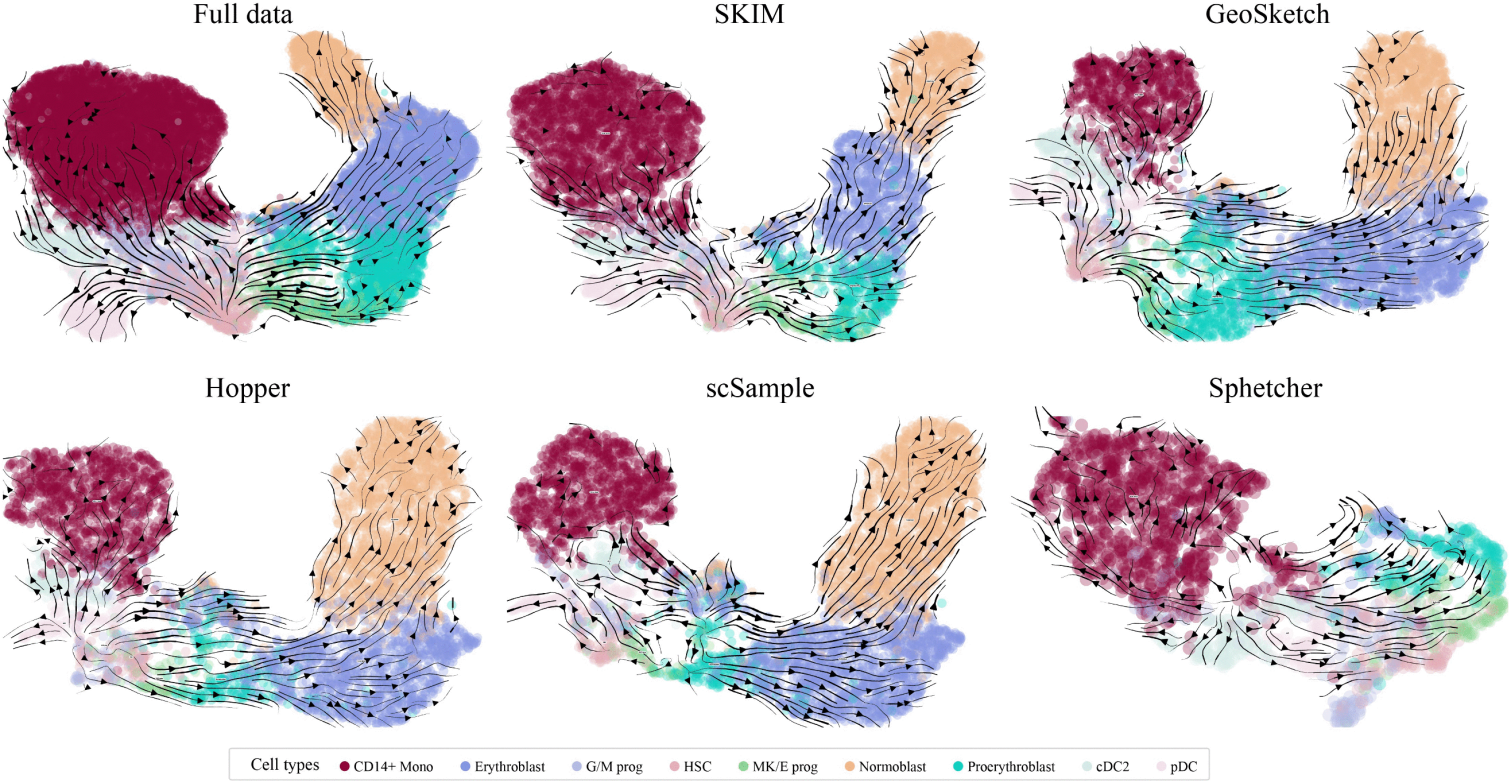
Developmental trajectory inference on the Hematopoiesis dataset using sketches generated by different sketching methods.

As shown in Fig 4, sketches generated by SKIM most accurately recapitulated the developmental continua of the full dataset, preserving major lineage bifurcations and smooth differentiation transitions. This performance is due to SKIM’s size-aware sampling strategy based on dynamic-feedback, which reduces redundancy within large homogeneous clusters while ensuring that rare and biologically informative cells, including transitional populations, are effectively retained. These rare cells are critical because they occupy intermediate states that define continuous differentiation paths, and their retention enables more accurate reconstruction of subtle lineage branches. By capturing these critical rare cells, SKIM preserves intermediate populations and subtle branches, enabling more complete and continuous reconstruction of differentiation trajectories.

In comparison, GeoSketch captured principal trajectories but lacked some intermediate resolution, while sketches from Sphetcher, Hopper, and scSampler exhibited discontinuities or missing branches. Additionally, the distribution of cells from different sketching methods on the Hematopoiesis dataset, visualized with UMAP (shown in S2 Fig), further validates SKIM’s superior retention of rare transitional cells.

To quantitatively evaluate trajectory preservation beyond visual inspection, we additionally compared cell type–level transition structures between the full dataset and the sketched datasets using two complementary metrics: transition Kendall correlation and transition mean absolute error (transition MAE; see S3 Text for full definitions and calculation details). As summarized in Table 3, SKIM achieved the highest transition Kendall correlation (0.8508) and the lowest transition MAE (0.0039) among all evaluated methods, supporting its ability to preserve lineage progression structures and developmental transition patterns more faithfully than the baseline methods.

**Table 3:** Quantitative assessment of hematopoiesis trajectory preservation across sketching methods using two transition metrics (Kendall correlation and mean absolute error).

	Transition Kendall	Transition MAE
SKIM	0.8508	0.0039
GeoSketch	0.7949	0.0105
Hopper	0.8172	0.0118
scSampler	0.8024	0.0123
Sphetcher	0.7212	0.0221

These results demonstrate that SKIM’s ability to retain rare and transitional cells provides a clear advantage in modeling developmental progression and yields more faithful lineage reconstruction than alternative methods.

### Evaluating computational efficiency and distributional consistency of sketching methods to the full dataset

To assess the computational efficiency and consistency of sketches in representing the full data distribution, sketching methods were benchmarked across six single-cell datasets spanning a wide range of sizes and complexities, from moderate-scale datasets such as PBMC (30,495 cells) to large-scale datasets such as Gut (428,469 cells). Each method generated a 10% sketch for every dataset to ensure a comparable evaluation. The similarity between each sketch and its corresponding full dataset was quantified using the Wasserstein distance, with lower values indicating closer alignment, while runtime measurements assessed computational efficiency. The detailed Wasserstein distance calculation is provided in S4 Text.

For fair benchmarking, runtime measurements excluded shared PCA preprocessing and file loading steps for all methods, since PCA representations served as the common input for GeoSketch, Hopper, Sphetcher, scSampler, and SKIM. Runtime therefore quantified the complete post-PCA sketch construction pipeline for each method. For SKIM, this included dynamic-feedback extractor training, reconstruction-loss trajectory extraction, filtering, FAISS-accelerated clustering, sampling, and final sketch construction. A detailed runtime decomposition of the SKIM pipeline is provided in S8 Table.

**Fig 5:**
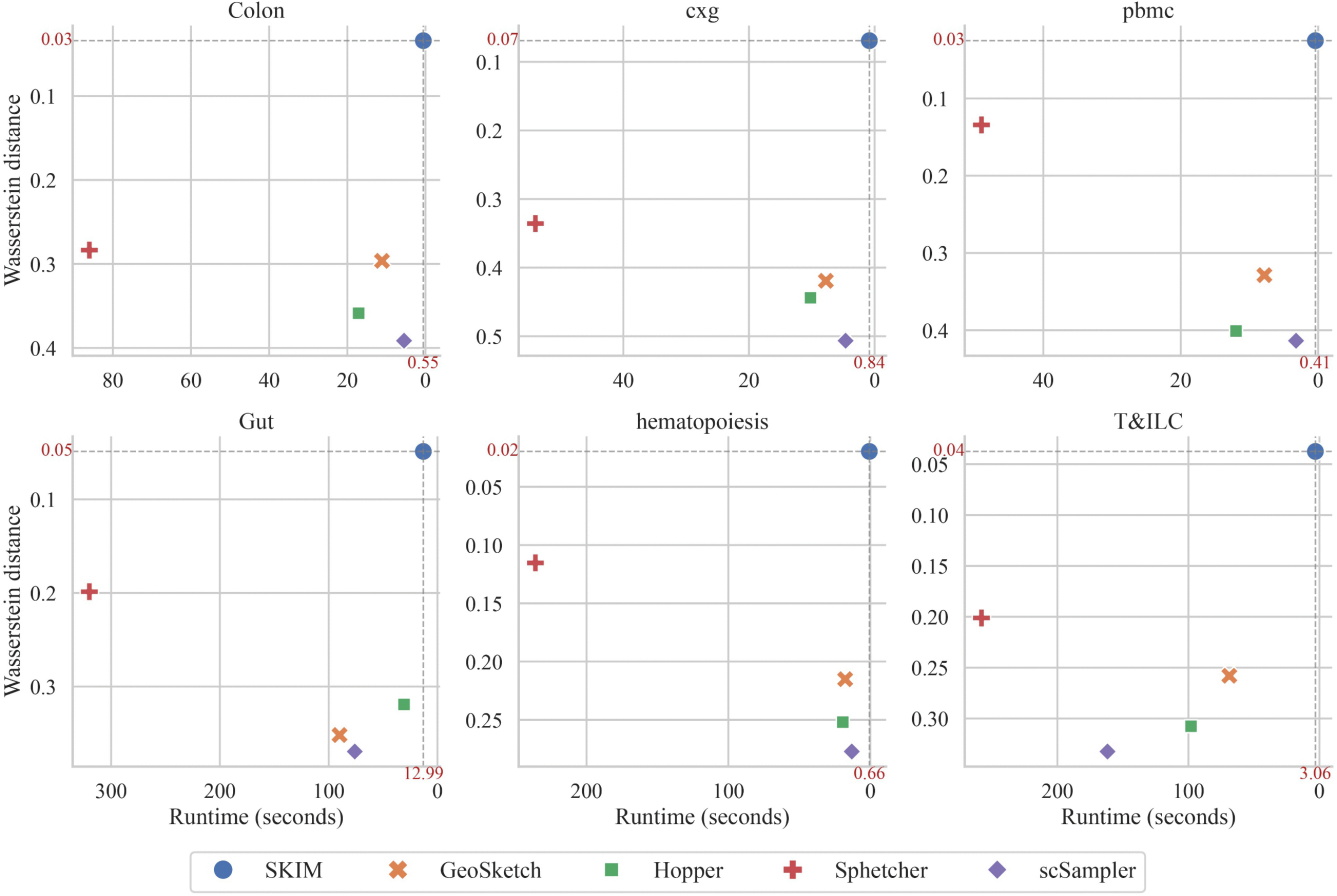
Benchmarking of computational efficiency and distributional consistency for SKIM and baseline sketching methods across six single-cell datasets.

As shown in Fig 5, SKIM achieved the lowest Wasserstein distances, indicating the most faithful preservation of the full data distribution. Other methods exhibited higher distances, suggesting less faithful representation. Runtime comparisons further highlighted SKIM’s efficiency: constructing a 10% sketch from the T&ILC dataset (216,611 cells) required only 3.06 seconds, over 20 times faster than existing approaches. In addition, peak memory usage was evaluated using resident set size (S3 Fig), showing that SKIM maintained relatively low memory consumption across datasets.

These results demonstrate that SKIM maintains both high fidelity and computational efficiency across datasets of varying sizes. Its combination of low Wasserstein distance and fast runtime underscores its scalability and practical utility, enabling rapid, representative sketching for downstream analyses such as cell type annotation, trajectory inference, and data integration.

### Evaluating hyperparameter robustness of SKIM via ablation analysis

To further evaluate the robustness and scalability of SKIM and the impact of its key hyperparameters, we conducted an ablation study using cell type classification on both the PBMC and Gut datasets as the downstream evaluation. CellTypist was used as the classifier, and sketches were generated from the training set at sampling ratios ranging from 2% to 10%. Two critical parameters were systematically varied: the filtering ratio, tuned from 0 to 0.05 to determine the proportion of abnormal cells to exclude, and the number of clusters in the k-means step, adjusted to assess the effect of clustering granularity. Additional ablation experiments exploring other hyperparameters can be found in S4 Fig.

**Fig 6:**
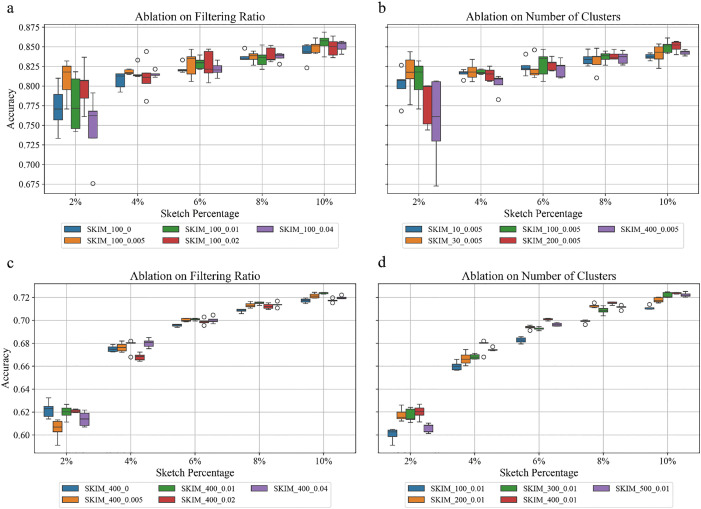
Hyperparameter robustness of SKIM assessed by downstream cell type classification accuracy. Effect of varying the filtering ratio on the PBMC (a) and Gut datasets (c). Effect of varying the number of clusters on the PBMC (b) and Gut datasets (d). Each model is denoted as SKIM_k_r, where k indicates the number of clusters used in k-means, and r represents the filtering ratio.

As shown in Fig 6a, for the PBMC dataset (30,495 cells), filtering ratios of 0.005 and 0.01 effectively removed uninformative cells without affecting rare but biologically relevant populations. Ratios above 0.02 began to exclude informative cells, leading to modest reductions in classification accuracy. As illustrated in Fig 6b, classification accuracy increased slightly as the number of clusters increased from 10 to 100, while increasing clusters beyond 100 yielded minimal additional benefit. This indicates that moderate clustering granularity is sufficient to capture diverse dynamic-feedback patterns.

We further performed the same ablation analysis on the Gut dataset (428,469 cells), which is substantially larger and more heterogeneous than PBMC. As shown in Fig 6c, both filtering ratios of r = 0.005 and r = 0.01 achieved comparable classification performance, demonstrating that SKIM is robust to the choice of filtering ratio in this complex dataset. In contrast, the number of clusters had a more scale-dependent effect. Classification accuracy improved when the number of clusters was increased from 100 to 400 on the Gut dataset (Fig 6d), likely because larger and more heterogeneous datasets contain more subtle and diverse dynamic-feedback patterns that can be better resolved with finer clustering granularity.

Collectively, these results indicate that SKIM is robust to the choice of filtering ratio, whereas the optimal number of clusters should be chosen in a scale- and heterogeneity-aware manner, with larger and more complex datasets benefiting from finer clustering granularity.

### Evaluating the characteristics of cells removed by dynamic-feedback-based filtering

To verify that the r-quantile filtering preferentially removes abnormal cells, we compared the retained and removed cells on the PBMC dataset using three independent doublet detection methods: Scrublet [24], scDblFinder[25], and DoubletFinder [26]. These tools estimate the likelihood that a cell is a doublet or contains mixed transcriptomic profiles, providing independent evidence for whether the removed cells are enriched for abnormal cell states.

**Fig 7:**
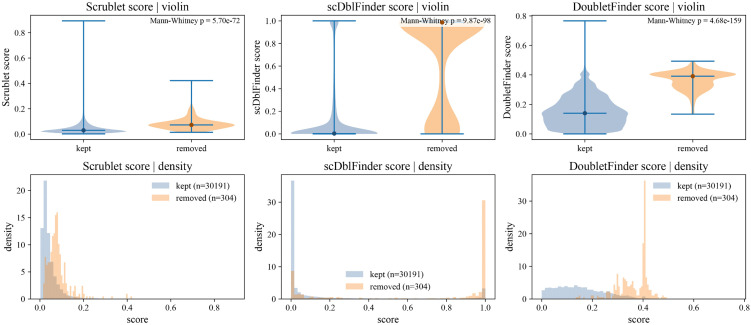
Distribution of doublet-associated scores for retained and removed cells in the PBMC dataset.

As shown in Fig 7, the distributions of doublet-associated scores differed substantially between retained and removed cells across all three methods. Both the violin plots and density distributions revealed a clear shift toward higher doublet-associated scores in the removed population. Specifically, the median Scrublet score increased from 0.030 in retained cells to 0.073 in removed cells, the median scDblFinder score increased from 0.004 to 0.986, and the median DoubletFinder pANN score increased from 0.140 to 0.391. These differences were highly significant according to two-sided Mann–Whitney U tests.

The substantial increase in doublet-associated scores among removed cells indicates that the r-quantile filtering step preferentially removes cells exhibiting technical-artifact-like signatures. These results provide empirical support for the effectiveness of the dynamic-feedback-based filtering strategy in identifying and excluding potentially artifactual cells.

## Discussion

In summary, we present SKIM, an efficient sketching framework for large-scale single-cell RNA-seq datasets. By leveraging dynamic-feedback computed from reconstruction losses across multiple training epochs, SKIM characterizes each cell from multiple perspectives, thereby capturing features from global expression patterns to local variations. Through clustering and size-aware sampling in the dynamic-feedback space, SKIM constructs representative sketches that reduce dataset size, balance population imbalances, and exclude abnormal cells, providing a compact yet biologically informative representation of large-scale single-cell datasets.

Extensive benchmarking across multiple downstream tasks demonstrates that SKIM effectively preserves essential biological information, including cell identity, population structure, and rare transitional states. These benefits arise from its multi-epoch dynamic-feedback, which ensures that both abundant and rare populations are accurately represented, providing the basis for its strong performance across cell type annotation, data integration, bulk RNA-seq deconvolution, and developmental trajectory inference. In both cell type annotation and cross-dataset cell type annotation tasks, SKIM maintains high accuracy, confirming that key cell identity signals are retained. For data integration, SKIM sketches better preserve global cellular structure, facilitating more effective batch mixing by downstream integration methods. For bulk RNA-seq deconvolution, sketches generated by SKIM capture representative transcriptomic expression profiles, allowing downstream deconvolution methods to reliably estimate cell-type proportions from bulk RNA-seq. During developmental trajectory inference, SKIM retains rare transitional cells, supporting faithful reconstruction of lineage progression and dynamic cellular transitions. Across all benchmarks, SKIM substantially reduces computational cost, generating a 10% sketch from 216,611 cells over 20 times faster than existing methods, while preserving essential biological information for downstream analyses.

Despite its strong performance, SKIM has several limitations. First, it currently focuses on transcriptomic data, and applying it to multi-modal single-cell datasets, such as spatial transcriptomics, proteomics, or epigenomics, may require adaptation of the feedback extraction and sketching procedures. Second, SKIM relies solely on reconstruction loss as the measure of model feedback; future extensions could incorporate additional sources of feedback, such as contrastive learning signals or information derived from graph-based relationships between cells. Future investigations could assess these factors to potentially expand SKIM’s applicability and improve performance on increasingly complex single-cell datasets.

Overall, SKIM provides a fast SKetching strategy Integrated with Model’s dynamic-feedback for large-scale single-cell transcriptomic analysis, enabling efficient and accurate downstream analyses of large-scale single-cell datasets.

## Method

### Overview

**Fig 8:**
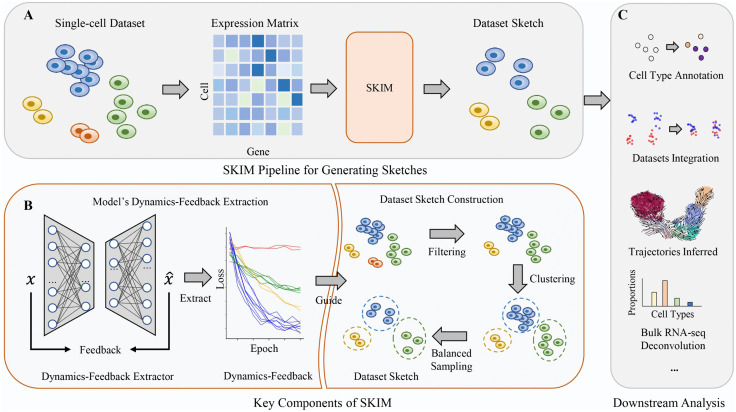
The SKIM framework generates compact and biologically representative sketches of large-scale single-cell datasets. (A) SKIM pipeline for generating sketches of large-scale single-cell datasets. (B) Key components of SKIM: Model’s Dynamics-Feedback Extraction and Dataset Sketch Construction (C) Example downstream tasks enabled by SKIM.

In this work, we introduce SKIM, a fast SKetching strategy Integrated with Model’s dynamic-feedback for large-scale single-cell transcriptomic analysis. SKIM leverages dynamic-feedback derived from reconstruction losses across multiple training epochs, capturing the progressive learning of each cell from global expression patterns to local variations. This enables the identification of abnormal cells and guides the construction of biologically representative sketches. The method operates in two main stages, as outlined in Fig 8:

1. Model’s Dynamic-Feedback Extraction: For each cell, reconstruction losses are recorded across multiple epochs of self-supervised training, with each epoch corresponding to a distinct set of model parameters. The resulting sequence of losses constitutes the dynamic-feedback, which reflects how the model progressively learns the transcriptomic expression profile. Even within dominant populations, cells with subtle transcriptomic differences generate distinct dynamic-feedback trajectories, capturing multiscale variation between cells. These trajectories allow SKIM to identify and preserve biologically informative cells, including rare or transitional populations, that would otherwise be obscured by abundant similar cells.
2. Dataset Sketch Construction: Following dynamic-feedback extraction, SKIM constructs the final dataset sketch by using the feedback to guide cell selection. Abnormal cells exhibiting minimal or stagnant decreases in reconstruction loss are filtered out to prevent distortion of downstream analyses. The remaining cells are clustered in the dynamic-feedback space, and a size-aware sampling strategy selects cells proportionally from each cluster. Small clusters containing unique or rare biological signals are prioritized, while larger clusters are downsampled to reduce redundancy. This ensures that both abundant and rare cell populations are adequately represented in the final sketch.

By integrating dynamic-feedback extraction, abnormal cell removal, and size-aware sampling, SKIM effectively constructs compact and biologically informative dataset sketches, enabling efficient and accurate downstream analyses.

### Data preprocessing

The raw single-cell expression data were preprocessed by normalization, log-transformation, and feature selection. Specifically, the data were normalized to a fixed total count, log-transformed, and the top 3,000 highly variable genes (HVGs) were selected. Principal component analysis (PCA) was then applied to reduce dimensionality to the top p components for downstream analysis (the detailed process can be found in S1 Text).

### Model’s dynamic-feedback extraction

Existing sketching methods focus on global expression patterns and often overlook local variations that encode multiscale transcriptomic differences, such as transitional or intermediate cell states, which are essential for preserving the full biological landscape. To address this limitation, SKIM leverages dynamic-feedback recorded across multiple training epochs. This feedback reflects how each cell is progressively learned by the model from global expression patterns to local variation, providing multiscale views of transcriptomic differences between cells. These properties enable SKIM to identify biologically informative cells, including rare or transitional populations, and guide the construction of compact and representative dataset sketches.

To extract dynamic-feedback, a lightweight dynamic-feedback extractor is constructed based on a self-supervised learning framework. Specifically, the extractor is implemented as a single-layer linear autoencoder consisting of a linear encoder and a linear decoder without activation functions. The latent dimensionality is set to 32. The model is optimized using Adam with a learning rate of 0.001 and trained for 15 epochs. We use default weight initialization in PyTorch. The detailed network architecture and training configuration are summarized in S9 Table. Each cell $x_{i}\in R^{p}$, where p denotes the number of dimensions after PCA, is first encoded into a latent representation $z_{i}$, which is then reconstructed as ${\hat{x}}_{i}$ by decoder. The reconstruction loss at epoch t is defined as


$$\begin{array}{c} L_{i}^{\left(t\right)}=\frac{\text{1}}{p}\sum_{j=\text{1}}^{p}||x_{ij}-{\hat{x}}_{ij}{\left|\right|}_{\text{2}}^{\text{2}}. \end{array}$$ (1)

Rather than considering only the final reconstruction loss, its evolution is recorded at regular sampling intervals during training. Let E denote the total number of training epochs and T denote the sampling stride. The sampled epoch set is defined as $S_{l}=\{t_{\text{1}},t_{\text{2}}...t_{m}\}$, where $m=\lfloor \frac{E}{T}\rfloor$. Sampling at these intervals captures how each cell contributes to model learning from early global patterns to later fine-grained variations, providing a multiscale view of transcriptomic differences beyond what a single final loss can convey. For each cell $x_{i}$, a feedback vector is formed as


$$\begin{array}{c} l_{i}=[L_{i}^{t_{\text{1}}},L_{i}^{t_{\text{2}}}...L_{i}^{t_{m}}]\in R^{m}. \end{array}$$ (2)

All cells’ feedback vectors form the feedback matrix:


$$\begin{array}{c} L=[\begin{array}{c} l_{\text{1}}\\ l_{\text{2}}\\ ...\\ l_{N} \end{array}]\in R^{N\times m}, \end{array}$$ (3)

where N is the total number of cells in the dataset. This dynamic-feedback therefore serves as a compact, multiscale descriptor of each cell, which is subsequently used to guide dataset sketch construction.

### Dataset sketch construction based on dynamic-feedback

Dynamic-feedback provides a principled representation of how cells are learned by the model across training, capturing meaningful transcriptomic variation while revealing abnormal cells. Cells identified as abnormal, typically arising from technical artifacts or low-quality measurements, exhibit unstable feedback trajectories and are removed to prevent distortion of downstream analyses. The remaining cells are clustered in the dynamic-feedback space, and a size-aware sampling strategy is applied to construct the final sketch. Small clusters, which often correspond to rare or transitional populations, are fully preserved, whereas larger clusters are proportionally downsampled to reduce redundancy, yielding a compact and biologically representative dataset sketch for downstream analyses.

First, SKIM identifies abnormal cells by quantifying each cell’s feedback decrement, which measures the change in reconstruction loss over training. For each cell i, the feedback decrement $\Delta L_{i}$ is defined as:


$$\begin{array}{c} \Delta L_{i}=L_{i}^{\left(t_{\text{1}}\right)}-L_{i}^{\left(t_{m}\right)}, \end{array}$$ (4)

where $L_{i}^{\left(t\right)}$ denotes the reconstruction loss of cell i at epoch t. Abnormal cells, characterized by minimal or inconsistent feedback decrements, contribute little meaningful information, deviate from the true data distribution, and can confound downstream analyses. In contrast, rare but biologically informative cells, although low in abundance, exhibit coherent and reproducible feedback decrements across training. This distinction allows SKIM to effectively separate rare, meaningful cells from abnormal ones during sketch construction. Abnormal cells are filtered using the r-quantile of all feedback decrements ${\left\{\Delta L_{i}\right\}}_{i=\text{1}}^{N}$, denoted as $Q_{r}({\left\{\Delta L_{i}\right\}}_{i=\text{1}}^{N})$. The set of filtered, informative cells F is then defined as


$$\begin{array}{c} F=\{i|\Delta L_{i}>Q_{r}({\left\{\Delta L_{i}\right\}}_{i=\text{1}}^{N})\}. \end{array}$$ (5)

After filtering, the remaining cells F are clustered based on their feedback to group cells with similar dynamic-feedback, using FAISS-accelerated k-means. A size-aware sampling strategy is employed to construct a compact sketch of size n, preferentially preserving smaller clusters while proportionally downsampling larger clusters. Clusters are sorted in ascending order of cluster size and processed sequentially so that smaller clusters are considered first. Let j∈{1,...,K} denote the cluster index after size-based sorting, where K is the total number of clusters. Each cluster $C_{j}$ contains $\left|C_{j}\right|$ cells. For each cluster $C_{j}$, the sampling quota is defined as


$$\begin{array}{c} n_{j}=min(|C_{j}|,\;\lfloor \frac{b_{j}}{K-j+\text{1}}\rfloor ), \end{array}$$ (6)

where $b_{j}$ denotes the remaining sampling budget before processing cluster $C_{j}$. The budget is updated iteratively as


$$\begin{array}{c} b_{j+\text{1}}=b_{j}-n_{j},\;b_{\text{1}}=n, \end{array}$$ (7)

where n is the target sketch size.

The final sketch S is obtained by combining selected subsets from all clusters:


$$\begin{array}{c} S={⋃}_{j}S_{j}, \end{array}$$ (8)

where $S_{j}$ denotes the selected cells from cluster $C_{j}$. This procedure ensures that the resulting sketch retains representative cells capturing both global expression patterns and local transcriptomic variation, including rare and transitional populations, while remaining compact and balanced. The sketch size n can be adjusted to accommodate computational constraints or downstream analysis requirements.

## Supporting information

S1 Table Model hyperparameters. (DOCX)

S2 Table Comparison of ACTINN classification accuracy across sketches of the CxG_min. (DOCX)

S3 Table Comparison of CellTypist classification accuracy across sketches of the CxG_min. (DOCX)

S4 Table Comparison of ACTINN classification accuracy across sketches of the PBMC. (DOCX)

S5 Table Comparison of CellTypist classification accuracy across Sketches of the PBMC. (DOCX)

S6 Table Integration performance of sketching methods using Scanorama across the ICA and Mammary_Gland dataset pairs. (DOCX)

S7 Table Integration performance of sketching methods using Harmony across the ICA and Mammary_Gland dataset pairs. (DOCX)

S8 Table Detailed runtime breakdown of SKIM across sketch construction stages on the T&ILC dataset. (DOCX)

S9 Table Architecture and training configuration of the dynamic-feedback extractor. (DOCX)

S1 Fig Predicted versus true cell-type proportions for each sketching strategy on the T&ILC. (DOCX)

S2 Fig Distribution of cells from different sketching methods on the Hematopoiesis visualized with UMAP. (DOCX)

S3 Fig Peak memory usage (peak RSS) of sketching methods across six single-cell datasets at a 10% sampling ratio. (DOCX)

S4 FigHyperparameter robustness of SKIM on the PBMC dataset assessed by downstream CellTypist classification accuracy.**(a) Effect of varying the number of sampled epochs** E**. (b) Effect of varying the sampling stride** T**. (c) Effect of varying the latent dimension of the autoencoder bottleneck.**(DOCX)

S5 Fig Sensitivity and runtime analysis of SKIM under different autoencoder architectures across sketch percentages. (DOCX)

S1 Text Data preprocessing. (DOCX)

S2 Text Detailed description of datasets used in this study. (DOCX)

S3 Text Quantitative evaluation of trajectory preservation. (DOCX)

S4 Text Wasserstein distance calculation. (DOCX)
