# Supplementary material for: SKIM: A fast sketching strategy integrated with model’s dynamic-feedback for large-scale single-cell transcriptomic analysis
Source: PLoS Comput Biol. 2026 Aug 10;22(8):e1014638. doi: 10.1371/journal.pcbi.1014638 (PMC13485126; doi:10.1371/journal.pcbi.1014638)
Supplement: Supplementary file 1 [file pcbi.1014638.s001.docx]

## S1 Table. Model Hyperparameter

| Parameters | Description | Default Value |
| --- | --- | --- |
| $p$ | Number of principal components retained after PCA | 50 |
| $r$ | Filtering quantile to remove cells with minimal loss decrements | 0.005 |
| $E$ | Total number of training epochs | 15 |
| $T$ | The sampling stride between consecutive recorded epochs | 3 |
| $K$ | Total number of clusters obtained via k-means | 100 |
| $d$ | Dimension of the latent representation | 32 |
