# Supplementary material for: SKIM: A fast sketching strategy integrated with model’s dynamic-feedback for large-scale single-cell transcriptomic analysis
Source: PLoS Comput Biol. 2026 Aug 10;22(8):e1014638. doi: 10.1371/journal.pcbi.1014638 (PMC13485126; doi:10.1371/journal.pcbi.1014638)
Supplement: Supplementary file 2 [file pcbi.1014638.s002.docx]

## S2 Table. Comparison of ACTINN Classification Accuracy Across Sketches of the CxG_min

| method | 2% | 4% | 6% | 8% | 10% |
| --- | --- | --- | --- | --- | --- |
| GeoSketch | 0.506±0.009 | 0.543±0.026 | 0.539±0.013 | 0.576±0.014 | 0.588±0.017 |
| Hopper | 0.497±0.048 | 0.508±0.024 | 0.567±0.018 | 0.576±0.022 | 0.580±0.020 |
| Sphetcher | 0.482±0.034 | 0.518±0.018 | 0.545±0.028 | 0.569±0.029 | 0.569±0.019 |
| scSampler | 0.550±0.032 | 0.540±0.033 | 0.540±0.032 | 0.575±0.033 | 0.584±0.029 |
| SKIM | 0.561±0.016 | 0.568±0.040 | 0.616±0.014 | 0.635±0.023 | 0.648±0.011 |

*Note: The accuracy on the full reference dataset was 0.7610.*
