# Supplementary material for: SKIM: A fast sketching strategy integrated with model’s dynamic-feedback for large-scale single-cell transcriptomic analysis
Source: PLoS Comput Biol. 2026 Aug 10;22(8):e1014638. doi: 10.1371/journal.pcbi.1014638 (PMC13485126; doi:10.1371/journal.pcbi.1014638)
Supplement: Supplementary file 3 [file pcbi.1014638.s003.docx]

## S3 Table. Comparison of CellTypist Classification Accuracy Across Sketches of the CxG_min

| method | 2% | 4% | 6% | 8% | 10% |
| --- | --- | --- | --- | --- | --- |
| GeoSketch | 0.433±0.016 | 0.445±0.009 | 0.504±0.010 | 0.534±0.009 | 0.574±0.008 |
| Hopper | 0.464±0.014 | 0.459±0.011 | 0.522±0.014 | 0.549±0.012 | 0.555±0.010 |
| Sphetcher | 0.477±0.015 | 0.514±0.010 | 0.548±0.024 | 0.552±0.012 | 0.563±0.006 |
| scSampler | 0.468±0.010 | 0.448±0.011 | 0.539±0.010 | 0.542±0.008 | 0.557±0.005 |
| SKIM | 0.517±0.010 | 0.520±0.010 | 0.567±0.011 | 0.574±0.006 | 0.589±0.007 |

*Note: The accuracy on the full reference dataset was 0.7102.*
