# Supplementary material for: SKIM: A fast sketching strategy integrated with model’s dynamic-feedback for large-scale single-cell transcriptomic analysis
Source: PLoS Comput Biol. 2026 Aug 10;22(8):e1014638. doi: 10.1371/journal.pcbi.1014638 (PMC13485126; doi:10.1371/journal.pcbi.1014638)
Supplement: Supplementary file 4 [file pcbi.1014638.s004.docx]

## S4 Table. Comparison of ACTINN Classification Accuracy Across Sketches of the PBMC

| method | 2% | 4% | 6% | 8% | 10% |
| --- | --- | --- | --- | --- | --- |
| GeoSketch | 0.748±0.047 | 0.791±0.015 | 0.798±0.011 | 0.813±0.013 | 0.823±0.007 |
| Hopper | 0.668±0.090 | 0.714±0.066 | 0.735±0.075 | 0.733±0.052 | 0.800±0.013 |
| Sphetcher | 0.794±0.021 | 0.822±0.010 | 0.826±0.006 | 0.840±0.006 | 0.841±0.028 |
| scSampler | 0.358±0.083 | 0.648±0.073 | 0.733±0.072 | 0.734±0.077 | 0.756±0.080 |
| SKIM | 0.800±0.014 | 0.809±0.010 | 0.833±0.007 | 0.855±0.008 | 0.850±0.006 |

*Note: The accuracy on the full reference dataset was 0.8943.*
