# Supplementary material for: SKIM: A fast sketching strategy integrated with model’s dynamic-feedback for large-scale single-cell transcriptomic analysis
Source: PLoS Comput Biol. 2026 Aug 10;22(8):e1014638. doi: 10.1371/journal.pcbi.1014638 (PMC13485126; doi:10.1371/journal.pcbi.1014638)
Supplement: Supplementary file 5 [file pcbi.1014638.s005.docx]

## S5 Table. Comparison of CellTypist Classification Accuracy Across Sketches of the PBMC

| method | 2% | 4% | 6% | 8% | 10% |
| --- | --- | --- | --- | --- | --- |
| GeoSketch | 0.626±0.055 | 0.811±0.017 | 0.795±0.034 | 0.834±0.011 | 0.829±0.017 |
| Hopper | 0.520±0.043 | 0.628±0.051 | 0.667±0.040 | 0.749±0.045 | 0.792±0.018 |
| Sphetcher | 0.792±0.016 | 0.825±0.006 | 0.822±0.004 | 0.818±0.004 | 0.828±0.003 |
| scSampler | 0.436±0.063 | 0.584±0.023 | 0.680±0.044 | 0.719±0.039 | 0.757±0.043 |
| SKIM | 0.798±0.026 | 0.820±0.003 | 0.819±0.007 | 0.847±0.005 | 0.852±0.007 |

*Note: The accuracy on the full reference dataset was 0.8186.*
