# Supplementary material for: SKIM: A fast sketching strategy integrated with model’s dynamic-feedback for large-scale single-cell transcriptomic analysis
Source: PLoS Comput Biol. 2026 Aug 10;22(8):e1014638. doi: 10.1371/journal.pcbi.1014638 (PMC13485126; doi:10.1371/journal.pcbi.1014638)
Supplement: Supplementary file 6 [file pcbi.1014638.s006.docx]

## S6 Table. Integration performance of sketching methods using Scanorama across the ICA and Mammary_Gland dataset pairs.

|  | ICA | | Mammary_Gland | |
| --- | --- | --- | --- | --- |
|  | ASW_label | iLISI | ASW_label | iLISI |
| GeoSketch | 0.6499 | 0.0117 | 0.4468 | 0.2136 |
| Hopper | 0.6525 | 0.0121 | 0.4466 | 0.2358 |
| Sphetcher | 0.6571 | 0.0288 | 0.4463 | 0.2482 |
| scSampler | 0.6430 | 0.0076 | 0.4465 | 0.2580 |
| SKIM | 0.6579 | 0.0457 | 0.4466 | 0.2557 |
| Full | 0.6588 | 0.0283 | 0.4458 | 0.2582 |
