# Supplementary material for: SKIM: A fast sketching strategy integrated with model’s dynamic-feedback for large-scale single-cell transcriptomic analysis
Source: PLoS Comput Biol. 2026 Aug 10;22(8):e1014638. doi: 10.1371/journal.pcbi.1014638 (PMC13485126; doi:10.1371/journal.pcbi.1014638)
Supplement: Supplementary file 7 [file pcbi.1014638.s007.docx]

## S7 Table. Integration performance of sketching methods using Harmony across the ICA and Mammary_Gland dataset pairs.

|  | ICA | | Mammary_Gland | |
| --- | --- | --- | --- | --- |
|  | ASW_label | iLISI | ASW_label | iLISI |
| GeoSketch | 0.4893 | 0.1654 | 0.5787 | 0.0149 |
| Hopper | 0.4885 | 0.1569 | 0.5529 | 0.0111 |
| Sphetcher | 0.4981 | 0.2106 | 0.5160 | 0.0059 |
| scSampler | 0.4887 | 0.1712 | 0.5616 | 0.0112 |
| SKIM | 0.5035 | 0.2006 | 0.5900 | 0.0752 |
| Full | 0.5041 | 0.6101 | 0.5946 | 0.1030 |
