# Supplementary material for: SKIM: A fast sketching strategy integrated with model’s dynamic-feedback for large-scale single-cell transcriptomic analysis
Source: PLoS Comput Biol. 2026 Aug 10;22(8):e1014638. doi: 10.1371/journal.pcbi.1014638 (PMC13485126; doi:10.1371/journal.pcbi.1014638)
Supplement: Supplementary file 8 [file pcbi.1014638.s008.docx]

## S8 Table. Detailed runtime breakdown of SKIM across sketch construction stages on the T&ILC dataset

| Stage | Runtime (seconds) |
| --- | --- |
| Model training and loss extraction | 2.608 |
| Filtering | 0.048 |
| FAISS clustering | 0.093 |
| Sampling | 0.249 |
| Output construction | 0.062 |
| Total accounted runtime | 3.061 |
