# Supplementary material for: SKIM: A fast sketching strategy integrated with model’s dynamic-feedback for large-scale single-cell transcriptomic analysis
Source: PLoS Comput Biol. 2026 Aug 10;22(8):e1014638. doi: 10.1371/journal.pcbi.1014638 (PMC13485126; doi:10.1371/journal.pcbi.1014638)
Supplement: Supplementary file 9 [file pcbi.1014638.s009.docx]

## S9 Table. Architecture and training configuration of the dynamic-feedback extractor

| Component | Configuration |
| --- | --- |
| Architecture | Linear Autoencoder |
| Encoder | Fully connected linear layer (PCA dim → 32) |
| Decoder | Fully connected linear layer (32 → PCA dim) |
| Activation function | None |
| Latent dimension | 32 |
| Optimizer | Adam |
| Learning rate for optimizer | 0.001 |
| Number of training epochs | 15 |
| Initialization | Default weight initialization in PyTorch |
| Learning rate for optimizer | 0.001 |
