# Supplementary material for: SKIM: A fast sketching strategy integrated with model’s dynamic-feedback for large-scale single-cell transcriptomic analysis
Source: PLoS Comput Biol. 2026 Aug 10;22(8):e1014638. doi: 10.1371/journal.pcbi.1014638 (PMC13485126; doi:10.1371/journal.pcbi.1014638)
Supplement: Supplementary file 10 [file pcbi.1014638.s010.docx]

## S1 Fig. Predicted versus true cell-type proportions for each sketching strategy on the T&ILC

*Note:Each subplot corresponds to a single cell type, with the x-axis representing the sketching method and the y-axis showing the ratio of predicted to true proportion. Bubble size reflects the number of samples. These plots illustrate that SKIM more accurately recapitulates bulk composition across all cell types compared to other sketching strategies.*
