# Supplementary material for: SKIM: A fast sketching strategy integrated with model’s dynamic-feedback for large-scale single-cell transcriptomic analysis
Source: PLoS Comput Biol. 2026 Aug 10;22(8):e1014638. doi: 10.1371/journal.pcbi.1014638 (PMC13485126; doi:10.1371/journal.pcbi.1014638)
Supplement: Supplementary file 12 [file pcbi.1014638.s012.docx]

## S3 Fig. Peak memory usage (peak RSS) of sketching methods across six single-cell datasets at a 10% sampling ratio.
