# Supplementary material for: SKIM: A fast sketching strategy integrated with model’s dynamic-feedback for large-scale single-cell transcriptomic analysis
Source: PLoS Comput Biol. 2026 Aug 10;22(8):e1014638. doi: 10.1371/journal.pcbi.1014638 (PMC13485126; doi:10.1371/journal.pcbi.1014638)
Supplement: Supplementary file 13 [file pcbi.1014638.s013.docx]

## S4 Fig. Hyperparameter robustness of SKIM on the PBMC dataset assessed by downstream CellTypist classification accuracy. (a) Effect of varying the number of sampled epochs $\boldsymbol{E}$ with fixed sampling stride $\boldsymbol{T=3}$. (b) Effect of varying the sampling stride $\boldsymbol{T}$ with fixed sampled epochs $\boldsymbol{E}\mathbf{=15}$. (c) Effect of varying the latent dimension of the autoencoder bottleneck. Boxplots summarize performance across sketch ratios ranging from 2% to 10%. Boxplots summarize performance across sketch ratios ranging from 2% to 10%.
