# Supplementary material for: SKIM: A fast sketching strategy integrated with model’s dynamic-feedback for large-scale single-cell transcriptomic analysis
Source: PLoS Comput Biol. 2026 Aug 10;22(8):e1014638. doi: 10.1371/journal.pcbi.1014638 (PMC13485126; doi:10.1371/journal.pcbi.1014638)
Supplement: Supplementary file 15 [file pcbi.1014638.s015.docx]

## S1 Text. Data Preprocessing

Raw single-cell expression data are processed using a standardized preprocessing pipeline, which comprises normalization, log-transformation, feature selection, and dimensionality reduction. The expression profile of each cell is scaled to a fixed total count $c_{0}$ to correct for differences in sequencing depth:

$$x_{ig}^{'}=\frac{x_{ig}}{\sum_{g^{'}=1}^{d} x_{ig^{'}}}\times c_{0}$$

where $x_{ig}$ denotes the raw count of gene $g$ in cell $i$, $x_{ig}^{'}$ is the normalized expression value, and $c_{0}={10}^{4}$ in this study. A log-transformation is then applied to stabilize variance:

$$x_{ig}^{''}=log(1+x_{ig}^{'})$$

where$x_{ig}^{''}$ is the log-transformed expression value for gene $g$ in cell $i$. This log-transformation helps stabilize the variance, especially for genes with highly skewed distributions.

To identify the top 3000 highly variable genes (HVGs), the scanpy.pp.highly_variable_genes function is applied. This function models the relationship between the mean and variance of the log-transformed gene expression values $x_{ig}^{''}$ across all genes and selects those with the highest standardized dispersion. The output is a reduced expression matrix $X_{HVG}\in R^{N*3000}$, where each row represents a cell and each column corresponds to one of the highly variable genes.

Principal Component Analysis (PCA) is then applied to $X_{HVG}$ to capture the principal axes of variation and reduce dimensionality, retaining the top $p=50$ components. The resulting reduced $\tilde{X}\in\mathbb{R}^{N*p}$ is used as input for SKIM.
