# Supplementary material for: SKIM: A fast sketching strategy integrated with model’s dynamic-feedback for large-scale single-cell transcriptomic analysis
Source: PLoS Comput Biol. 2026 Aug 10;22(8):e1014638. doi: 10.1371/journal.pcbi.1014638 (PMC13485126; doi:10.1371/journal.pcbi.1014638)
Supplement: Supplementary file 16 [file pcbi.1014638.s016.docx]

## S2 Text. Detailed description of datasets used in this study

To evaluate the performance of SKIM across a broad range of downstream single-cell analysis tasks, we used eight publicly available scRNA-seq datasets covering immune profiling, intestinal atlases, hematopoietic systems, and developmental processes. These datasets differ substantially in size, tissue origin, annotation resolution, and batch complexity, allowing us to assess whether sketching preserves biologically meaningful information under diverse conditions.

The **PBMC** dataset was used for cell type annotation within a well-characterized immune-cell setting. It contains 30,495 cells and 10 annotated cell types. Because this dataset is widely used as a reference benchmark for immune-cell classification, it provides a controlled setting for evaluating whether sketches retain cell identity information.

The **CxG** dataset corresponds to the minimal training and test subsets from the scTab benchmark and is derived from CELLxGENE resources. Importantly, it is not a single study, but a highly heterogeneous multi-study compendium assembled from many independent experiments. The training subset contains 32,768 cells spanning 233 source datasets, 2,889 donors, 7 assay categories, 170 tissues, 53 broad tissue groups, 47 disease states, 143 developmental stages, and 164 cell types. The test subset contains 32,768 cells spanning 136 source datasets and 162 cell types. This high degree of biological and technical diversity makes CxG a particularly demanding benchmark for testing whether sketches preserve information needed for robust cell type annotation across heterogeneous reference data.

The **Colon** dataset was used together with the Gut dataset for cross-dataset cell type annotation. It contains 41,650 cells and 25 annotated cell types collected from multiple donors across four anatomical regions. This dataset represents a structured immune-cell atlas of the human colon and serves as one side of the cross-dataset annotation benchmark.

The **Gut** dataset was used as the paired target dataset for cross-dataset annotation and is the largest dataset in this study, containing 428,469 cells and 134 annotated cell types. It includes cells from multiple diagnostic groups and anatomical regions, with substantial batch diversity. Its high complexity makes it suitable for testing whether sketching preserves cell identity across different biological conditions and technical backgrounds.

The **ica cord blood** dataset was used in the data integration benchmark. It contains 384,000 cells with broad hematopoietic and immune composition, making it useful for testing whether sketches preserve the global structure required for effective downstream integration.

The **ica bone marrow** dataset was paired with the cord blood dataset for the data integration task. It contains 378,000 cells and a wide range of hematopoietic cell states, providing a complementary setting for evaluating whether sketching preserves structures necessary for integration across sources.

The **T&ILC** dataset was used for bulk RNA-seq deconvolution. It contains 216,611 cells from multiple donors, tissues, and assay types, with 16 annotated immune-cell populations. Its focused immune composition and broad tissue sampling make it suitable for testing whether sketches preserve representative expression profiles required for accurate deconvolution.

The **hematopoiesis** dataset was used for developmental trajectory inference. It contains 67,568 cells collected across multiple sites, donors, and batches, spanning 12 annotated cell types. This dataset is well suited for assessing whether sketching retains the continuity of developmental structure.

The **Mammary_Gland** dataset was used for same-tissue, cross-technology integration. It contains 15,577 cells and 7 annotated cell types, combining droplet-based and FACS-based scRNA-seq profiles from the Tabula Muris Senis resource. Because the two subsets are derived from the same tissue but generated using different profiling technologies, this dataset provides a controlled setting for evaluating whether sketching preserves the biological structure required for integration while reducing confounding from tissue-level biological differences.
