# Supplementary material for: SKIM: A fast sketching strategy integrated with model’s dynamic-feedback for large-scale single-cell transcriptomic analysis
Source: PLoS Comput Biol. 2026 Aug 10;22(8):e1014638. doi: 10.1371/journal.pcbi.1014638 (PMC13485126; doi:10.1371/journal.pcbi.1014638)
Supplement: Supplementary file 17 [file pcbi.1014638.s017.docx]

## S3 Text. Quantitative Evaluation of Trajectory Preservation

To quantitatively evaluate how well developmental trajectories were preserved after sketching, we compared cell type–level transition matrices inferred from the full dataset and corresponding sketched datasets using CellRank2. Let $M_{ij}$ denote the transition probability from cell type $i$ to cell type $j$. Diagonal self-transitions $M_{ii}$ were excluded to focus on developmental transitions between distinct cell types, and the remaining inter-cell-type transitions were vectorized for analysis.

Transition ordering consistency was quantified using the Kendall rank correlation coefficient:

$$\tau=Kendall(m^{f},m^{s})$$

where $m^{f}$ and $m^{s}$ are the vectorized inter-cell-type transitions from the full and sketched datasets, respectively. Higher values of $\tau$ indicate better preservation of the relative ordering of transition strengths, reflecting that the sketch preserves the hierarchical structure of lineage progression.

To assess absolute deviations in transition probabilities, we computed the transition mean absolute error (transition MAE):

$$MAE=\frac{1}{N_{pair}}\sum|m^{f}-m^{s}|$$

where $N_{pair}$ denotes the total number of inter-cell-type transitions. Lower MAE indicates that the sketch more accurately reproduces the magnitude of developmental transition probabilities observed in the full dataset.

Together, the transition Kendall correlation and transition MAE provide complementary evaluations of trajectory preservation, capturing both structural consistency and quantitative fidelity of developmental transitions in sketched datasets.
