# Supplementary material for: SKIM: A fast sketching strategy integrated with model’s dynamic-feedback for large-scale single-cell transcriptomic analysis
Source: PLoS Comput Biol. 2026 Aug 10;22(8):e1014638. doi: 10.1371/journal.pcbi.1014638 (PMC13485126; doi:10.1371/journal.pcbi.1014638)
Supplement: Supplementary file 18 [file pcbi.1014638.s018.docx]

## S4 Text. Wasserstein Distance calculation

To quantify the distributional similarity between each sketch and the corresponding full dataset, Wasserstein Distance was computed in the PCA embedding space. Let ${X_{\mathrm{full}}\mathbb{\in R}}^{N*p}$ and ${X_{sketch}\mathbb{\in R}}^{n*p}$ denote the PCA embeddings of the full and sketched datasets, respectively, where $N$ and $n$ are the numbers of cells in the full dataset and sketch, and $p$ is the number of retained principal components. For each PCA coordinate $j\in\{1,...,p\}$, we computed the one-dimensional Wasserstein Distance between the distributions of the full and sketched cells along that coordinate. The final Wasserstein Distance was defined as the average across all retained principal components:

$$WD=\frac{1}{p}\sum_{j=1}^{p} W_{1}({X_{\mathrm{full}}}_{.j},{X_{sketch}}_{.j}).$$
